# (8a*R*,9*R*)-9-Hy­droxy-7,8,8a,9-tetra­hydro­furo[3,2-*f*]indolizin-6(4*H*)-one

**DOI:** 10.1107/S1600536812040378

**Published:** 2012-09-29

**Authors:** Viktor Vrábel, Ľubomír Švorc, Peter Šafář, Július Sivý, Žúžiová Jozefína

**Affiliations:** a Institute of Analytical Chemistry, Faculty of Chemical and Food Technology, Slovak University of Technology, Radlinského 9, SK-812 37 Bratislava, Slovak Republic 81237; b Institute of Organic Chemistry, Catalysis and Petrochemistry, Faculty of Chemical and Food Technology, Slovak University of Technology, Radlinského 9, SK-812 37 Bratislava, Slovak Republic 81237; c Institute of Mathematics and Physics, Faculty of Mechanical Engineering STU, Námestie slobody 17, SK-812 37 Bratislava, Slovak Republic

## Abstract

The title compound, C_10_H_11_NO_3_, crystallizes with four independent mol­ecules in the asymmetric unit. Their geometries are very similar and corresponding bond distances are almost identical. The central six-membered ring of the indolizine moiety adopts a envelope conformation [the displacement of the flap atom (the C atom opposite the N atom) being 0.539 (2), 0.548 (3), 0.509 (3) and 0.544 (3) Å in the four molecules], while the conformation of the oxopyrrolidine ring is close to that of a flat envelope. The displacements of the non-fused C atom opposite the C=O group of the pyrrolidine ring of the four mol­ecules are 0.366 (3), 0.335 (3), 0.173 (3) and −0.310 (3) Å. In the crystal, O—H⋯O hydrogen bonds link the mol­ecules into chains, which run parallel to the *c* axis. The absolute configuration was assigned from the synthesis.

## Related literature

For background to indolizines and their biological activity, see: Gubin *et al.* (1992 ▶); Gundersen *et al.* (2007 ▶); Gupta *et al.* (2003 ▶); Mikael (1999 ▶); Pyne (2005 ▶); Teklu *et al.* (2005 ▶). For asymmetry parameters, see: Nardelli (1983 ▶).
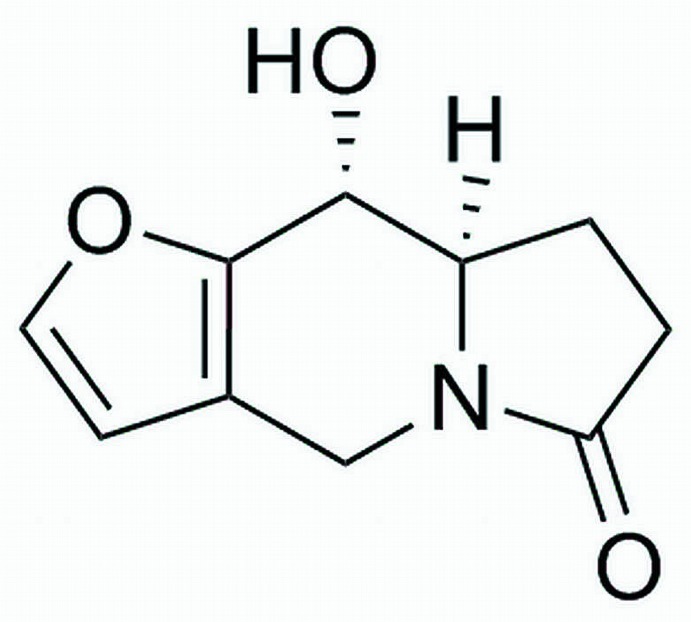


## Experimental

### 

#### Crystal data

C_10_H_11_NO_3_
*M* *_r_* = 193.20
Orthorhombic, 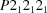

*a* = 14.7603 (10) Å
*b* = 15.1301 (17) Å
*c* = 16.2847 (9) Å
*V* = 3636.8 (5) Å^3^
*Z* = 16
Mo *K*α radiation
μ = 0.11 mm^−1^
*T* = 298 K
0.58 × 0.34 × 0.09 mm

#### Data collection

Oxford Diffraction Gemini R CCD diffractometer
Absorption correction: analytical (Clark & Reid, 1995 ▶) *T*_min_ = 0.953, *T*_max_ = 0.989
55202 measured reflections
3581 independent reflections
3168 reflections with *I* > 2σ(*I*)
*R*_int_ = 0.036

#### Refinement

*R*[*F* ^2^ > 2σ(*F* ^2^)] = 0.034
*wR*(*F* ^2^) = 0.086
*S* = 1.04
3581 reflections
522 parameters
4 restraints
H atoms treated by a mixture of independent and constrained refinement
Δρ_max_ = 0.17 e Å^−3^
Δρ_min_ = −0.15 e Å^−3^

### 

Data collection: *CrysAlis CCD* (Oxford Diffraction, 2006 ▶); cell refinement: *CrysAlis CCD*; data reduction: *CrysAlis RED* (Oxford Diffraction, 2006 ▶); program(s) used to solve structure: *SHELXS97* (Sheldrick, 2008 ▶); program(s) used to refine structure: *SHELXL97* (Sheldrick, 2008 ▶); molecular graphics: *DIAMOND* (Brandenburg, 2001 ▶); software used to prepare material for publication: *SHELXL97*, *PLATON* (Spek, 2009 ▶) and *WinGX* (Farrugia, 1999 ▶).

## Supplementary Material

Crystal structure: contains datablock(s) I, global. DOI: 10.1107/S1600536812040378/ds2219sup1.cif

Structure factors: contains datablock(s) I. DOI: 10.1107/S1600536812040378/ds2219Isup2.hkl

Supplementary material file. DOI: 10.1107/S1600536812040378/ds2219Isup3.cml

Additional supplementary materials:  crystallographic information; 3D view; checkCIF report

## Figures and Tables

**Table 1:** Hydrogen-bond geometry (Å, °)

*D*—H⋯*A*	*D*—H	H⋯*A*	*D*⋯*A*	*D*—H⋯*A;*
O3—H3*O*⋯O2^i^	0.84 (2)	1.90 (2)	2.681 (2)	154 (3)
O6—H6*O*⋯O8^ii^	0.86 (2)	1.93 (2)	2.766 (3)	166 (4)
O9—H9*O*⋯O5^ii^	0.85 (2)	1.92 (2)	2.737 (3)	161 (3)
O12—H12*O*⋯O11^iii^	0.83 (2)	1.98 (2)	2.797 (3)	167 (3)

Symmetry codes: (i) 
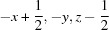
; (ii) 
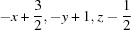
; (iii) 
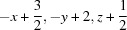
.

**Fig. 1.**
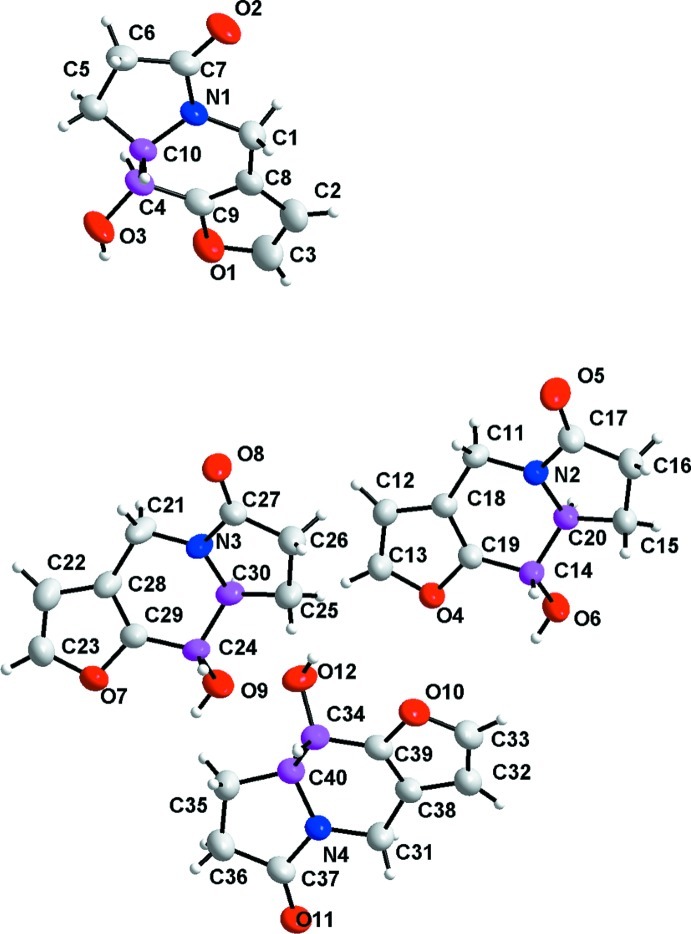
Molecular structure of (I) with the atomic numbering scheme of the four independent molecules. Displacement ellipsoids are drawn at the 50% probability level (Brandenburg, 2001).

**Fig. 2.**
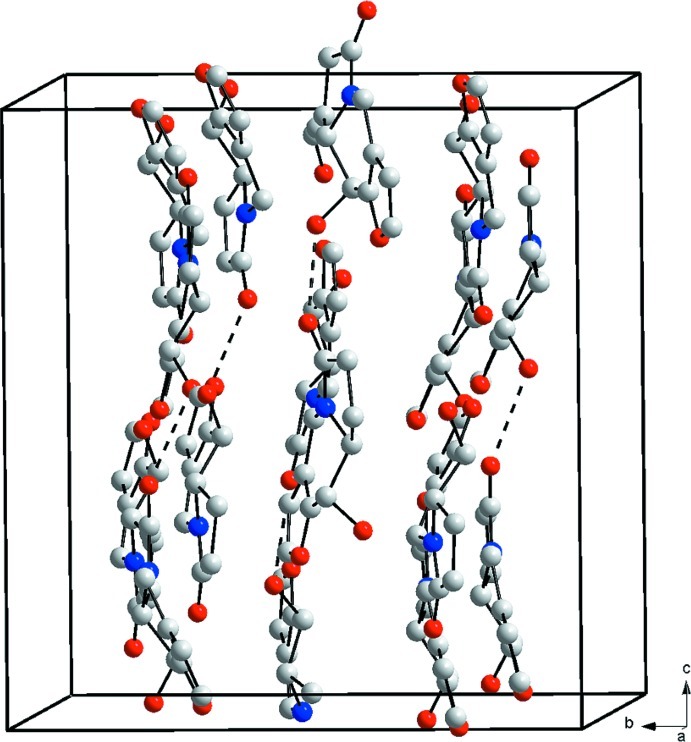
Packing view of (I) and showing the formation of the hydrogen-bonded chains running along the c axis. H atoms have been omitted for clarity.

## supplementary crystallographic information

### Comment

Indolizines are electron-rich heterocycles with very low oxidation potential. Functionalized indolizines are common substructures found in biologically important natural products and synthetic pharmaceuticals. Due to the various biological functions associated with this skeleton, it has been frequently employed as a key scaffold in the drug industry (Gundersen *et al.*, 2007). Indolizine alkaloids are excellent inhibitors of biologically important pathways. These include the binding and processing of glycoproteins, potent glycosidase inhibitory activities (Pyne, 2005), activity against AIDS virus HIV and some carcinogenic cells (Mikael, 1999). They have also shown to be calcium entry blockers (Gupta *et al.*, 2003) and potent antioxidants inhibiting lipid peroxidation *in vitro* (Teklu *et al.*, 2005). As such, indolizines are important synthetic targets in view of developing new pharmaceuticals for the treatment of cardiovascular diseases (Gubin *et al.*, 1992). Based on these facts and in contitutation of our interest in developing simple and efficient route for the synthesis of novel indolizine derivatives. The molecular structure and the atom labeling scheme of four independent molecules in the asymmetric unit are shown in Fig. 1. The absolute configuration was established by synthesis and is depicted in the scheme and Fig.1. The expected stereochemistry of atoms C4 and C10, C14 and C20, C24 and C30, C34 and C40 in the four molecules was confirmed as *R*, *R*, respectively (Fig. 1). The central six-membered rings of the four molecules according to Nardelli (Nardelli, 1983) is not planar and adopts an envelope conformation, with atoms C10, C20, C30 and C40 as the flaps. The displacements of atoms C10, C20, C30 and C40 from the mean planes of the remaining five atoms are 0.539 (2), 0.548 (3), 0.509 (3) and 0.544 (3) Å, respectively. The bond lengths of the carbonyl groups C=O in the four molecules are somewhat longer than typical carbonyl bonds. This may be due to the fact that atoms O2, O5, O8 and O11 participates in intermolecular hydrogen bonds, they seem to be effective in the stabilization of the structure. The central six-membered rings form dihedral angles of 21.8 (1), 20.1 (1), 24.9 (1) and 22.5 (1)° with the oxopyrrolidine rings, in the four independent molecules, respectively. Intermolecular O–H···O hydrogen bonds link the molecules into extended chains, which run parallel to the *c* axis (Fig.2).

### Experimental

The title compound (8aR,*RS*)-9-hydroxy-7,8,8a,9-tetrahydrofuro[3,2-*f*] indolizin-6(4*H*)-one was prepared by a reduction of (*R*)-8,8a-dihydrofuro[3,2-*f*]indolizine-6,9(4*H*,7H)-dione with sodium borohydride. To a solution of a freshly crystallized keto-lactam (191 mg, 1 mmoL) in methanol (10 ml) was added in a small portions sodium borohydride (42 mg, 1,1 mmoL) at 0–5°C. The mixture was then stirred at 0°C for 10 h, until total disappearance of starting materials was observed (TLC). The solution was carefully neutralized with 36% HCl, and the solvent was removed under vacuum. The obtained solution was then extracted with dichloromethane (3 *x* 25 ml). The organic layer was dried over MgSO4, concentrated *in vacuo* to afford a colourless oil, which quickly crystallized on standing in a fridge. Recrystallization from n-hexane/ethylacetate (9:1) gave colourless crystals of the title compound (150 mg, 78%).

### Refinement

All H atoms bonded to C atoms were placed in geometrically idealized positions and constrained to ride on their parent atoms, with C—H distances in the range 0.93 - 0.98 Å and *U*_iso_ set at 1.2*U*_eq_ of the parent atom. H atoms of the hidroxyl groups were located in a difference map and finally refined with O—H distance fixed at 0.84 Å. In the absence of significant anomalous scattering, Friedel pairs were merged and the absolute configuration was assigned from the starting material.

### Crystal data

**Table:**

C_10_H_11_NO_3_	*F*(000) = 1632
*M**_r_* = 193.20	*D*_x_ = 1.411 Mg m^−^^3^
Orthorhombic, *P*2_1_2_1_2_1_	Mo *K*α radiation, λ = 0.71073 Å
Hall symbol: P 2ac 2ab	Cell parameters from 13239 reflections
*a* = 14.7603 (10) Å	θ = 2.3–29.5°
*b* = 15.1301 (17) Å	µ = 0.11 mm^−^^1^
*c* = 16.2847 (9) Å	*T* = 298 K
*V* = 3636.8 (5) Å^3^	Prism, colourless
*Z* = 16	0.58 × 0.34 × 0.09 mm

### Data collection

**Table:**

Oxford Diffraction Gemini R CCD diffractometer	3581 independent reflections
Radiation source: fine-focus sealed tube	3168 reflections with *I* > 2σ(*I*)
Graphite monochromator	*R*_int_ = 0.036
Detector resolution: 10.4340 pixels mm^-1^	θ_max_ = 25.0°, θ_min_ = 3.0°
Rotation method data acquisition using ω and φ scans	*h* = −17→17
Absorption correction: analytical (Clark & Reid, 1995)	*k* = −18→18
*T*_min_ = 0.953, *T*_max_ = 0.989	*l* = −18→19
55202 measured reflections	

### Refinement

**Table:**

Refinement on *F*^2^	Primary atom site location: structure-invariant direct methods
Least-squares matrix: full	Secondary atom site location: difference Fourier map
*R*[*F*^2^ > 2σ(*F*^2^)] = 0.034	Hydrogen site location: inferred from neighbouring sites
*wR*(*F*^2^) = 0.086	H atoms treated by a mixture of independent and constrained refinement
*S* = 1.04	*w* = 1/[σ^2^(*F*_o_^2^) + (0.0452*P*)^2^ + 0.6847*P*] where *P* = (*F*_o_^2^ + 2*F*_c_^2^)/3
3581 reflections	(Δ/σ)_max_ < 0.001
522 parameters	Δρ_max_ = 0.17 e Å^−^^3^
4 restraints	Δρ_min_ = −0.15 e Å^−^^3^

### Special details

**Table:**

Geometry. All e.s.d.'s (except the e.s.d. in the dihedral angle between two l.s. planes) are estimated using the full covariance matrix. The cell e.s.d.'s are taken into account individually in the estimation of e.s.d.'s in distances, angles and torsion angles; correlations between e.s.d.'s in cell parameters are only used when they are defined by crystal symmetry. An approximate (isotropic) treatment of cell e.s.d.'s is used for estimating e.s.d.'s involving l.s. planes.
Refinement. Refinement of *F*^2^ against ALL reflections. The weighted *R*-factor *wR* and goodness of fit *S* are based on *F*^2^, conventional *R*-factors *R* are based on *F*, with *F* set to zero for negative *F*^2^. The threshold expression of *F*^2^ > σ(*F*^2^) is used only for calculating *R*-factors(gt) *etc*. and is not relevant to the choice of reflections for refinement. *R*-factors based on *F*^2^ are statistically about twice as large as those based on *F*, and *R*- factors based on ALL data will be even larger.

### Fractional atomic coordinates and isotropic or equivalent isotropic displacement parameters (Å^2^)

**Table:**

	*x*	*y*	*z*	*U*_iso_*/*U*_eq_	Occ. (<1)
C1	0.37091 (18)	−0.0319 (2)	0.96600 (15)	0.0505 (7)	
H1B	0.3935	−0.0811	0.9982	0.061*	
H1A	0.4014	0.0215	0.9841	0.061*	
C2	0.4725 (2)	−0.0647 (2)	0.83402 (17)	0.0564 (8)	
H2	0.5299	−0.0706	0.8569	0.068*	
C3	0.4509 (2)	−0.0705 (2)	0.75510 (19)	0.0687 (9)	
H3	0.4924	−0.0805	0.7131	0.082*	
C4	0.22570 (19)	−0.02846 (19)	0.83141 (14)	0.0460 (6)	
H4	0.1929	−0.0848	0.8307	0.055*	
C5	0.12006 (19)	0.0081 (2)	0.95377 (15)	0.0493 (7)	
H5B	0.0837	0.0598	0.9415	0.059*	
H5A	0.0889	−0.0441	0.9336	0.059*	
C6	0.1382 (2)	0.0008 (2)	1.04619 (15)	0.0552 (8)	
H6B	0.1293	0.0574	1.0730	0.066*	
H6A	0.0985	−0.0425	1.0713	0.066*	
C7	0.2351 (2)	−0.02766 (19)	1.05206 (15)	0.0475 (7)	
C8	0.38974 (18)	−0.04746 (18)	0.87672 (14)	0.0435 (6)	
C9	0.32426 (19)	−0.04454 (19)	0.81992 (14)	0.0458 (6)	
C10	0.21457 (17)	0.01593 (17)	0.91544 (14)	0.0401 (6)	
H10	0.2296	0.0787	0.9099	0.048*	
C11	0.85665 (16)	0.2162 (2)	0.71399 (14)	0.0440 (6)	
H11A	0.8232	0.2569	0.7486	0.053*	
H11B	0.8399	0.1564	0.7293	0.053*	
C12	0.74899 (17)	0.2316 (2)	0.58353 (16)	0.0467 (6)	
H12	0.6925	0.2181	0.6054	0.056*	
C13	0.76729 (17)	0.2542 (2)	0.50606 (17)	0.0524 (7)	
H13	0.7242	0.2586	0.4646	0.063*	
C14	0.99586 (16)	0.26724 (19)	0.58311 (14)	0.0424 (6)	
H14	1.0086	0.3302	0.5914	0.051*	
C15	1.11005 (17)	0.2440 (2)	0.70238 (15)	0.0488 (7)	
H15A	1.1594	0.2057	0.6856	0.059*	
H15B	1.1256	0.3046	0.6888	0.059*	
C16	1.09142 (17)	0.2346 (2)	0.79415 (15)	0.0510 (7)	
H16A	1.1189	0.1811	0.8155	0.061*	
H16B	1.1153	0.2849	0.8241	0.061*	
C17	0.99049 (17)	0.2306 (2)	0.80141 (14)	0.0454 (6)	
C18	0.83367 (16)	0.23212 (18)	0.62607 (14)	0.0392 (6)	
C19	0.89690 (16)	0.25498 (17)	0.57055 (13)	0.0391 (6)	
C20	1.02126 (16)	0.21675 (18)	0.66021 (14)	0.0397 (6)	
H20	1.0246	0.1537	0.6468	0.048*	
C21	0.38642 (19)	0.6723 (3)	0.70964 (15)	0.0648 (9)	
H21B	0.3492	0.6994	0.7518	0.078*	
H21A	0.3754	0.6091	0.7102	0.078*	
C22	0.27775 (19)	0.7229 (2)	0.58750 (17)	0.0563 (8)	
H22	0.2209	0.7086	0.6082	0.068*	
C23	0.29579 (18)	0.7594 (2)	0.51509 (18)	0.0544 (7)	
H23	0.2522	0.7751	0.4765	0.065*	
C24	0.52638 (17)	0.74252 (19)	0.58671 (14)	0.0446 (6)	
H24	0.5454	0.8036	0.5970	0.054*	
C25	0.63795 (18)	0.7101 (2)	0.70505 (15)	0.0539 (7)	
H25B	0.6854	0.6679	0.6925	0.065*	
H25A	0.6580	0.7685	0.6883	0.065*	
C26	0.61646 (19)	0.7090 (2)	0.79567 (16)	0.0577 (8)	
H26B	0.6483	0.6611	0.8228	0.069*	
H26A	0.6340	0.7643	0.8211	0.069*	
C27	0.51560 (17)	0.69575 (19)	0.80162 (14)	0.0435 (6)	
C28	0.36308 (17)	0.7098 (2)	0.62739 (14)	0.0477 (7)	
C29	0.42635 (16)	0.73930 (18)	0.57528 (14)	0.0416 (6)	
C30	0.55016 (17)	0.68515 (18)	0.66060 (14)	0.0402 (6)	
H30	0.5549	0.6236	0.6423	0.048*	
C31	0.88814 (18)	0.9793 (2)	0.52818 (16)	0.0516 (7)	
H31A	0.9201	1.0281	0.5025	0.062*	
H31B	0.9082	0.9248	0.5027	0.062*	
C32	0.99215 (19)	0.9749 (2)	0.66191 (17)	0.0540 (7)	
H32	1.0500	0.9713	0.6395	0.065*	
C33	0.9707 (2)	0.9787 (2)	0.74150 (17)	0.0605 (8)	
H33	1.0127	0.9786	0.7841	0.073*	
C34	0.74269 (18)	0.98715 (19)	0.66276 (14)	0.0427 (6)	
H34	0.7229	1.0486	0.6695	0.051*	
C35	0.63366 (19)	0.9893 (2)	0.53834 (17)	0.0559 (8)	
H35B	0.5875	0.9439	0.5422	0.067*	
H35A	0.6126	1.0416	0.5670	0.067*	
C36	0.6542 (2)	1.0103 (3)	0.45011 (17)	0.0644 (9)	
H36B	0.6268	1.0660	0.4344	0.077*	
H36A	0.6312	0.9642	0.4143	0.077*	
C37	0.7559 (2)	1.0157 (2)	0.44446 (16)	0.0495 (7)	
C38	0.90826 (17)	0.97727 (18)	0.61805 (14)	0.0432 (6)	
C39	0.84266 (18)	0.98120 (18)	0.67511 (14)	0.0431 (6)	
C40	0.72346 (17)	0.95645 (18)	0.57500 (14)	0.0420 (6)	
H40	0.7242	0.8917	0.5736	0.050*	
N1	0.27316 (15)	−0.02275 (15)	0.97777 (12)	0.0438 (5)	
N2	0.95392 (13)	0.22898 (15)	0.72598 (11)	0.0394 (5)	
N3	0.48159 (14)	0.69005 (16)	0.72568 (11)	0.0445 (5)	
N4	0.79082 (14)	0.99003 (15)	0.51692 (12)	0.0443 (5)	
O1	0.35925 (14)	−0.05982 (16)	0.74337 (11)	0.0643 (6)	
O2	0.27650 (15)	−0.05083 (16)	1.11433 (10)	0.0666 (6)	
O3	0.18597 (15)	0.03090 (19)	0.77435 (11)	0.0726 (7)	
O4	0.85804 (12)	0.27000 (15)	0.49559 (10)	0.0526 (5)	
O5	0.94469 (13)	0.22702 (18)	0.86442 (10)	0.0684 (7)	
O6	1.04954 (13)	0.23539 (19)	0.51857 (11)	0.0707 (7)	
O7	0.38731 (13)	0.77089 (14)	0.50474 (11)	0.0534 (5)	
O8	0.46942 (13)	0.69031 (16)	0.86436 (10)	0.0597 (8)	0.997 (7)
O9	0.57598 (15)	0.7075 (2)	0.51995 (11)	0.0767 (8)	
O10	0.87864 (13)	0.98284 (15)	0.75233 (10)	0.0565 (5)	
O11	0.80178 (14)	1.03788 (18)	0.38476 (11)	0.0695 (7)	
O12	0.69166 (14)	0.93163 (16)	0.71545 (11)	0.0595 (6)	
H3O	0.2085 (19)	0.024 (2)	0.7273 (13)	0.063 (9)*	
H6O	1.034 (3)	0.260 (3)	0.4732 (16)	0.096 (13)*	
H9O	0.557 (2)	0.726 (2)	0.4740 (14)	0.080 (11)*	
H12O	0.702 (2)	0.944 (2)	0.7642 (12)	0.070 (10)*	

### Atomic displacement parameters (Å^2^)

**Table:**

	*U*^11^	*U*^22^	*U*^33^	*U*^12^	*U*^13^	*U*^23^
C1	0.0463 (16)	0.0713 (19)	0.0337 (13)	0.0001 (14)	−0.0075 (12)	0.0001 (13)
C2	0.0521 (17)	0.0692 (19)	0.0479 (16)	0.0176 (15)	−0.0010 (14)	−0.0049 (14)
C3	0.062 (2)	0.095 (3)	0.0485 (18)	0.0281 (19)	0.0087 (15)	−0.0073 (17)
C4	0.0547 (16)	0.0569 (17)	0.0265 (12)	0.0028 (14)	−0.0044 (12)	0.0008 (11)
C5	0.0492 (16)	0.0630 (19)	0.0356 (14)	0.0012 (14)	−0.0005 (12)	0.0059 (13)
C6	0.0585 (18)	0.074 (2)	0.0334 (13)	0.0039 (16)	0.0067 (13)	0.0082 (13)
C7	0.0586 (17)	0.0567 (17)	0.0270 (13)	0.0024 (14)	0.0016 (13)	0.0011 (11)
C8	0.0487 (14)	0.0469 (15)	0.0351 (13)	0.0049 (13)	−0.0013 (11)	0.0021 (11)
C9	0.0570 (16)	0.0523 (16)	0.0282 (12)	0.0073 (13)	0.0005 (12)	−0.0037 (12)
C10	0.0459 (15)	0.0437 (14)	0.0307 (12)	−0.0004 (12)	−0.0002 (11)	0.0046 (11)
C11	0.0381 (13)	0.0650 (18)	0.0290 (12)	−0.0092 (12)	0.0016 (11)	0.0032 (12)
C12	0.0360 (13)	0.0655 (17)	0.0385 (14)	−0.0063 (13)	−0.0021 (11)	0.0033 (13)
C13	0.0383 (13)	0.081 (2)	0.0377 (14)	−0.0044 (14)	−0.0066 (12)	0.0073 (14)
C14	0.0354 (13)	0.0597 (16)	0.0322 (12)	0.0023 (12)	0.0064 (10)	0.0050 (12)
C15	0.0366 (13)	0.0696 (19)	0.0402 (13)	0.0038 (13)	−0.0027 (11)	0.0058 (13)
C16	0.0423 (14)	0.074 (2)	0.0364 (13)	0.0063 (14)	−0.0072 (12)	−0.0018 (13)
C17	0.0441 (14)	0.0644 (18)	0.0276 (12)	0.0036 (13)	−0.0016 (11)	−0.0057 (12)
C18	0.0388 (13)	0.0489 (15)	0.0300 (12)	−0.0025 (12)	0.0002 (10)	0.0016 (11)
C19	0.0379 (13)	0.0526 (16)	0.0269 (11)	0.0010 (12)	−0.0013 (10)	0.0008 (11)
C20	0.0390 (13)	0.0499 (15)	0.0301 (12)	0.0048 (12)	0.0037 (10)	0.0016 (11)
C21	0.0465 (16)	0.120 (3)	0.0282 (13)	−0.0248 (18)	0.0024 (12)	0.0046 (15)
C22	0.0431 (15)	0.084 (2)	0.0418 (15)	−0.0075 (15)	0.0009 (13)	−0.0131 (15)
C23	0.0444 (15)	0.0708 (19)	0.0479 (16)	0.0022 (14)	−0.0079 (13)	0.0037 (15)
C24	0.0411 (14)	0.0574 (17)	0.0354 (13)	0.0020 (13)	0.0113 (11)	0.0114 (12)
C25	0.0425 (14)	0.080 (2)	0.0389 (14)	0.0026 (14)	0.0057 (12)	0.0023 (14)
C26	0.0455 (16)	0.091 (2)	0.0362 (14)	0.0039 (15)	0.0000 (12)	0.0066 (15)
C27	0.0448 (14)	0.0570 (17)	0.0286 (12)	0.0020 (13)	0.0011 (12)	0.0025 (12)
C28	0.0405 (14)	0.0725 (19)	0.0302 (12)	−0.0109 (14)	0.0048 (11)	−0.0070 (12)
C29	0.0426 (14)	0.0518 (15)	0.0303 (12)	−0.0005 (12)	0.0036 (11)	0.0008 (12)
C30	0.0437 (14)	0.0469 (15)	0.0299 (12)	−0.0004 (12)	0.0091 (11)	−0.0011 (11)
C31	0.0430 (15)	0.079 (2)	0.0326 (13)	0.0029 (15)	0.0030 (12)	−0.0012 (13)
C32	0.0477 (16)	0.0695 (19)	0.0447 (15)	−0.0013 (14)	−0.0052 (13)	0.0083 (14)
C33	0.0527 (18)	0.089 (2)	0.0401 (15)	−0.0074 (17)	−0.0116 (14)	0.0112 (15)
C34	0.0466 (15)	0.0508 (16)	0.0306 (12)	−0.0007 (12)	0.0057 (11)	−0.0020 (11)
C35	0.0421 (15)	0.081 (2)	0.0444 (15)	0.0020 (15)	−0.0015 (13)	0.0031 (15)
C36	0.0514 (17)	0.096 (3)	0.0458 (16)	0.0099 (17)	−0.0055 (14)	0.0108 (17)
C37	0.0520 (16)	0.0674 (19)	0.0291 (13)	0.0057 (14)	−0.0016 (12)	0.0018 (13)
C38	0.0450 (14)	0.0519 (16)	0.0327 (12)	0.0028 (13)	−0.0006 (11)	0.0034 (11)
C39	0.0502 (15)	0.0506 (15)	0.0286 (12)	0.0000 (13)	−0.0017 (12)	−0.0002 (11)
C40	0.0439 (14)	0.0519 (16)	0.0303 (12)	0.0004 (12)	0.0023 (11)	−0.0014 (11)
N1	0.0482 (12)	0.0587 (14)	0.0245 (10)	0.0018 (11)	−0.0029 (9)	0.0027 (10)
N2	0.0350 (11)	0.0571 (13)	0.0262 (10)	−0.0029 (10)	0.0009 (8)	−0.0008 (9)
N3	0.0382 (12)	0.0712 (15)	0.0241 (10)	−0.0083 (11)	0.0047 (9)	−0.0022 (10)
N4	0.0414 (12)	0.0622 (14)	0.0293 (10)	0.0036 (11)	0.0028 (10)	0.0040 (10)
O1	0.0657 (13)	0.0953 (17)	0.0320 (9)	0.0244 (13)	−0.0005 (9)	−0.0092 (10)
O2	0.0790 (14)	0.0961 (16)	0.0246 (9)	0.0203 (13)	−0.0032 (9)	0.0025 (10)
O3	0.0677 (14)	0.123 (2)	0.0269 (10)	0.0363 (14)	0.0015 (10)	0.0122 (11)
O4	0.0398 (9)	0.0880 (15)	0.0299 (8)	−0.0008 (10)	−0.0017 (8)	0.0116 (10)
O5	0.0515 (11)	0.127 (2)	0.0270 (9)	−0.0015 (13)	0.0019 (9)	−0.0075 (11)
O6	0.0462 (11)	0.134 (2)	0.0322 (10)	0.0249 (13)	0.0103 (9)	0.0193 (13)
O7	0.0500 (11)	0.0663 (13)	0.0440 (10)	0.0019 (10)	0.0007 (9)	0.0168 (10)
O8	0.0482 (12)	0.1068 (18)	0.0241 (10)	−0.0013 (11)	0.0046 (8)	−0.0012 (10)
O9	0.0595 (13)	0.140 (2)	0.0310 (10)	0.0296 (14)	0.0184 (10)	0.0243 (12)
O10	0.0582 (13)	0.0843 (15)	0.0270 (9)	−0.0078 (11)	−0.0046 (8)	0.0028 (9)
O11	0.0577 (12)	0.1188 (19)	0.0319 (10)	−0.0001 (13)	0.0015 (9)	0.0169 (11)
O12	0.0626 (12)	0.0833 (15)	0.0326 (10)	−0.0202 (11)	0.0071 (9)	−0.0007 (10)

### Geometric parameters (Å, º)

**Table:**

C1—N1	1.462 (3)	C21—H21B	0.9700
C1—C8	1.499 (3)	C21—H21A	0.9700
C1—H1B	0.9700	C22—C23	1.329 (4)
C1—H1A	0.9700	C22—C28	1.431 (4)
C2—C3	1.327 (4)	C22—H22	0.9300
C2—C8	1.429 (4)	C23—O7	1.372 (3)
C2—H2	0.9300	C23—H23	0.9300
C3—O1	1.376 (4)	C24—O9	1.414 (3)
C3—H3	0.9300	C24—C29	1.489 (3)
C4—O3	1.419 (3)	C24—C30	1.525 (3)
C4—C9	1.487 (4)	C24—H24	0.9800
C4—C10	1.533 (3)	C25—C26	1.509 (4)
C4—H4	0.9800	C25—C30	1.532 (4)
C5—C6	1.533 (4)	C25—H25B	0.9700
C5—C10	1.533 (4)	C25—H25A	0.9700
C5—H5B	0.9700	C26—C27	1.505 (4)
C5—H5A	0.9700	C26—H26B	0.9700
C6—C7	1.496 (4)	C26—H26A	0.9700
C6—H6B	0.9700	C27—O8	1.231 (3)
C6—H6A	0.9700	C27—N3	1.337 (3)
C7—O2	1.235 (3)	C28—C29	1.339 (3)
C7—N1	1.336 (3)	C29—O7	1.371 (3)
C8—C9	1.338 (3)	C30—N3	1.467 (3)
C9—O1	1.369 (3)	C30—H30	0.9800
C10—N1	1.456 (3)	C31—N4	1.457 (3)
C10—H10	0.9800	C31—C38	1.494 (3)
C11—N2	1.462 (3)	C31—H31A	0.9700
C11—C18	1.491 (3)	C31—H31B	0.9700
C11—H11A	0.9700	C32—C33	1.336 (4)
C11—H11B	0.9700	C32—C38	1.430 (4)
C12—C13	1.335 (4)	C32—H32	0.9300
C12—C18	1.429 (3)	C33—O10	1.371 (3)
C12—H12	0.9300	C33—H33	0.9300
C13—O4	1.371 (3)	C34—O12	1.417 (3)
C13—H13	0.9300	C34—C39	1.492 (4)
C14—O6	1.402 (3)	C34—C40	1.529 (3)
C14—C19	1.487 (3)	C34—H34	0.9800
C14—C20	1.517 (3)	C35—C36	1.502 (4)
C14—H14	0.9800	C35—C40	1.536 (4)
C15—C16	1.526 (3)	C35—H35B	0.9700
C15—C20	1.536 (4)	C35—H35A	0.9700
C15—H15A	0.9700	C36—C37	1.506 (4)
C15—H15B	0.9700	C36—H36B	0.9700
C16—C17	1.496 (4)	C36—H36A	0.9700
C16—H16A	0.9700	C37—O11	1.232 (3)
C16—H16B	0.9700	C37—N4	1.345 (3)
C17—O5	1.230 (3)	C38—C39	1.343 (3)
C17—N2	1.342 (3)	C39—O10	1.365 (3)
C18—C19	1.345 (3)	C40—N4	1.463 (3)
C19—O4	1.368 (3)	C40—H40	0.9800
C20—N2	1.473 (3)	O3—H3O	0.842 (18)
C20—H20	0.9800	O6—H6O	0.855 (19)
C21—N3	1.454 (3)	O9—H9O	0.848 (19)
C21—C28	1.494 (4)	O12—H12O	0.829 (18)
			
N1—C1—C8	109.0 (2)	C22—C23—H23	124.5
N1—C1—H1B	109.9	O7—C23—H23	124.5
C8—C1—H1B	109.9	O9—C24—C29	113.9 (2)
N1—C1—H1A	109.9	O9—C24—C30	105.9 (2)
C8—C1—H1A	109.9	C29—C24—C30	108.0 (2)
H1B—C1—H1A	108.3	O9—C24—H24	109.6
C3—C2—C8	106.1 (3)	C29—C24—H24	109.6
C3—C2—H2	126.9	C30—C24—H24	109.6
C8—C2—H2	126.9	C26—C25—C30	106.3 (2)
C2—C3—O1	111.3 (3)	C26—C25—H25B	110.5
C2—C3—H3	124.4	C30—C25—H25B	110.5
O1—C3—H3	124.4	C26—C25—H25A	110.5
O3—C4—C9	115.2 (2)	C30—C25—H25A	110.5
O3—C4—C10	105.2 (2)	H25B—C25—H25A	108.7
C9—C4—C10	106.8 (2)	C27—C26—C25	105.8 (2)
O3—C4—H4	109.8	C27—C26—H26B	110.6
C9—C4—H4	109.8	C25—C26—H26B	110.6
C10—C4—H4	109.8	C27—C26—H26A	110.6
C6—C5—C10	104.2 (2)	C25—C26—H26A	110.6
C6—C5—H5B	110.9	H26B—C26—H26A	108.7
C10—C5—H5B	110.9	O8—C27—N3	123.7 (2)
C6—C5—H5A	110.9	O8—C27—C26	127.6 (2)
C10—C5—H5A	110.9	N3—C27—C26	108.7 (2)
H5B—C5—H5A	108.9	C29—C28—C22	106.3 (2)
C7—C6—C5	104.5 (2)	C29—C28—C21	122.3 (2)
C7—C6—H6B	110.8	C22—C28—C21	131.5 (2)
C5—C6—H6B	110.8	C28—C29—O7	110.8 (2)
C7—C6—H6A	110.8	C28—C29—C24	128.6 (2)
C5—C6—H6A	110.8	O7—C29—C24	120.7 (2)
H6B—C6—H6A	108.9	N3—C30—C24	112.5 (2)
O2—C7—N1	123.4 (3)	N3—C30—C25	103.28 (18)
O2—C7—C6	127.4 (2)	C24—C30—C25	115.3 (2)
N1—C7—C6	109.2 (2)	N3—C30—H30	108.5
C9—C8—C2	106.7 (2)	C24—C30—H30	108.5
C9—C8—C1	122.1 (2)	C25—C30—H30	108.5
C2—C8—C1	131.2 (2)	N4—C31—C38	108.8 (2)
C8—C9—O1	110.6 (2)	N4—C31—H31A	109.9
C8—C9—C4	128.7 (2)	C38—C31—H31A	109.9
O1—C9—C4	120.8 (2)	N4—C31—H31B	109.9
N1—C10—C5	103.03 (18)	C38—C31—H31B	109.9
N1—C10—C4	112.5 (2)	H31A—C31—H31B	108.3
C5—C10—C4	115.3 (2)	C33—C32—C38	106.1 (3)
N1—C10—H10	108.6	C33—C32—H32	126.9
C5—C10—H10	108.6	C38—C32—H32	126.9
C4—C10—H10	108.6	C32—C33—O10	111.2 (2)
N2—C11—C18	109.3 (2)	C32—C33—H33	124.4
N2—C11—H11A	109.8	O10—C33—H33	124.4
C18—C11—H11A	109.8	O12—C34—C39	114.1 (2)
N2—C11—H11B	109.8	O12—C34—C40	106.7 (2)
C18—C11—H11B	109.8	C39—C34—C40	106.9 (2)
H11A—C11—H11B	108.3	O12—C34—H34	109.7
C13—C12—C18	106.3 (2)	C39—C34—H34	109.7
C13—C12—H12	126.9	C40—C34—H34	109.7
C18—C12—H12	126.9	C36—C35—C40	105.4 (2)
C12—C13—O4	111.1 (2)	C36—C35—H35B	110.7
C12—C13—H13	124.5	C40—C35—H35B	110.7
O4—C13—H13	124.5	C36—C35—H35A	110.7
O6—C14—C19	114.2 (2)	C40—C35—H35A	110.7
O6—C14—C20	107.9 (2)	H35B—C35—H35A	108.8
C19—C14—C20	107.1 (2)	C35—C36—C37	105.7 (2)
O6—C14—H14	109.2	C35—C36—H36B	110.6
C19—C14—H14	109.2	C37—C36—H36B	110.6
C20—C14—H14	109.2	C35—C36—H36A	110.6
C16—C15—C20	105.0 (2)	C37—C36—H36A	110.6
C16—C15—H15A	110.7	H36B—C36—H36A	108.7
C20—C15—H15A	110.7	O11—C37—N4	124.1 (3)
C16—C15—H15B	110.7	O11—C37—C36	127.7 (3)
C20—C15—H15B	110.7	N4—C37—C36	108.2 (2)
H15A—C15—H15B	108.8	C39—C38—C32	106.2 (2)
C17—C16—C15	105.1 (2)	C39—C38—C31	122.2 (2)
C17—C16—H16A	110.7	C32—C38—C31	131.5 (2)
C15—C16—H16A	110.7	C38—C39—O10	110.9 (2)
C17—C16—H16B	110.7	C38—C39—C34	128.5 (2)
C15—C16—H16B	110.7	O10—C39—C34	120.5 (2)
H16A—C16—H16B	108.8	N4—C40—C34	111.9 (2)
O5—C17—N2	122.8 (2)	N4—C40—C35	102.9 (2)
O5—C17—C16	128.0 (2)	C34—C40—C35	115.2 (2)
N2—C17—C16	109.2 (2)	N4—C40—H40	108.9
C19—C18—C12	106.4 (2)	C34—C40—H40	108.9
C19—C18—C11	122.0 (2)	C35—C40—H40	108.9
C12—C18—C11	131.6 (2)	C7—N1—C10	113.8 (2)
C18—C19—O4	110.6 (2)	C7—N1—C1	121.9 (2)
C18—C19—C14	128.4 (2)	C10—N1—C1	122.2 (2)
O4—C19—C14	120.9 (2)	C17—N2—C11	121.3 (2)
N2—C20—C14	111.8 (2)	C17—N2—C20	113.4 (2)
N2—C20—C15	102.53 (19)	C11—N2—C20	123.30 (19)
C14—C20—C15	116.5 (2)	C27—N3—C21	122.7 (2)
N2—C20—H20	108.6	C27—N3—C30	114.3 (2)
C14—C20—H20	108.6	C21—N3—C30	121.8 (2)
C15—C20—H20	108.6	C37—N4—C31	121.4 (2)
N3—C21—C28	108.3 (2)	C37—N4—C40	114.0 (2)
N3—C21—H21B	110.0	C31—N4—C40	123.4 (2)
C28—C21—H21B	110.0	C9—O1—C3	105.3 (2)
N3—C21—H21A	110.0	C4—O3—H3O	111 (2)
C28—C21—H21A	110.0	C19—O4—C13	105.65 (19)
H21B—C21—H21A	108.4	C14—O6—H6O	111 (3)
C23—C22—C28	106.5 (2)	C29—O7—C23	105.5 (2)
C23—C22—H22	126.8	C24—O9—H9O	112 (2)
C28—C22—H22	126.8	C39—O10—C33	105.4 (2)
C22—C23—O7	111.0 (2)	C34—O12—H12O	111 (2)
			
C8—C2—C3—O1	−1.1 (4)	C33—C32—C38—C39	−0.8 (3)
C10—C5—C6—C7	−19.8 (3)	C33—C32—C38—C31	176.7 (3)
C5—C6—C7—O2	−171.7 (3)	N4—C31—C38—C39	2.6 (4)
C5—C6—C7—N1	9.6 (4)	N4—C31—C38—C32	−174.6 (3)
C3—C2—C8—C9	0.2 (4)	C32—C38—C39—O10	0.9 (3)
C3—C2—C8—C1	−178.1 (3)	C31—C38—C39—O10	−176.8 (3)
N1—C1—C8—C9	5.4 (4)	C32—C38—C39—C34	178.4 (3)
N1—C1—C8—C2	−176.6 (3)	C31—C38—C39—C34	0.6 (5)
C2—C8—C9—O1	0.7 (3)	O12—C34—C39—C38	136.9 (3)
C1—C8—C9—O1	179.2 (3)	C40—C34—C39—C38	19.2 (4)
C2—C8—C9—C4	−179.8 (3)	O12—C34—C39—O10	−45.9 (4)
C1—C8—C9—C4	−1.4 (5)	C40—C34—C39—O10	−163.6 (2)
O3—C4—C9—C8	135.7 (3)	O12—C34—C40—N4	−163.2 (2)
C10—C4—C9—C8	19.3 (4)	C39—C34—C40—N4	−40.8 (3)
O3—C4—C9—O1	−44.9 (4)	O12—C34—C40—C35	79.8 (3)
C10—C4—C9—O1	−161.3 (2)	C39—C34—C40—C35	−157.8 (2)
C6—C5—C10—N1	22.5 (3)	C36—C35—C40—N4	19.1 (3)
C6—C5—C10—C4	145.4 (2)	C36—C35—C40—C34	141.1 (3)
O3—C4—C10—N1	−163.7 (2)	O2—C7—N1—C10	−173.1 (3)
C9—C4—C10—N1	−40.8 (3)	C6—C7—N1—C10	5.6 (3)
O3—C4—C10—C5	78.6 (3)	O2—C7—N1—C1	−9.3 (5)
C9—C4—C10—C5	−158.5 (2)	C6—C7—N1—C1	169.4 (3)
C18—C12—C13—O4	−0.5 (4)	C5—C10—N1—C7	−18.2 (3)
C20—C15—C16—C17	−16.8 (3)	C4—C10—N1—C7	−142.9 (2)
C15—C16—C17—O5	−176.4 (3)	C5—C10—N1—C1	178.0 (3)
C15—C16—C17—N2	5.2 (4)	C4—C10—N1—C1	53.3 (3)
C13—C12—C18—C19	−0.1 (3)	C8—C1—N1—C7	165.1 (3)
C13—C12—C18—C11	177.2 (3)	C8—C1—N1—C10	−32.4 (4)
N2—C11—C18—C19	−0.3 (4)	O5—C17—N2—C11	−4.6 (5)
N2—C11—C18—C12	−177.3 (3)	C16—C17—N2—C11	173.8 (3)
C12—C18—C19—O4	0.7 (3)	O5—C17—N2—C20	−168.9 (3)
C11—C18—C19—O4	−177.0 (2)	C16—C17—N2—C20	9.5 (3)
C12—C18—C19—C14	178.5 (3)	C18—C11—N2—C17	170.8 (3)
C11—C18—C19—C14	0.9 (5)	C18—C11—N2—C20	−26.5 (4)
O6—C14—C19—C18	140.8 (3)	C14—C20—N2—C17	−145.3 (2)
C20—C14—C19—C18	21.4 (4)	C15—C20—N2—C17	−19.8 (3)
O6—C14—C19—O4	−41.6 (4)	C14—C20—N2—C11	50.7 (3)
C20—C14—C19—O4	−161.0 (2)	C15—C20—N2—C11	176.3 (2)
O6—C14—C20—N2	−165.6 (2)	O8—C27—N3—C21	−3.2 (5)
C19—C14—C20—N2	−42.3 (3)	C26—C27—N3—C21	176.5 (3)
O6—C14—C20—C15	77.0 (3)	O8—C27—N3—C30	−171.4 (3)
C19—C14—C20—C15	−159.7 (2)	C26—C27—N3—C30	8.3 (3)
C16—C15—C20—N2	21.4 (3)	C28—C21—N3—C27	154.5 (3)
C16—C15—C20—C14	143.8 (2)	C28—C21—N3—C30	−38.1 (4)
C28—C22—C23—O7	0.2 (4)	C24—C30—N3—C27	−137.6 (3)
C30—C25—C26—C27	−7.4 (4)	C25—C30—N3—C27	−12.7 (3)
C25—C26—C27—O8	179.6 (3)	C24—C30—N3—C21	54.1 (4)
C25—C26—C27—N3	−0.1 (4)	C25—C30—N3—C21	179.0 (3)
C23—C22—C28—C29	−0.3 (3)	O11—C37—N4—C31	−5.3 (5)
C23—C22—C28—C21	179.4 (3)	C36—C37—N4—C31	173.6 (3)
N3—C21—C28—C29	10.5 (4)	O11—C37—N4—C40	−172.8 (3)
N3—C21—C28—C22	−169.1 (3)	C36—C37—N4—C40	6.1 (4)
C22—C28—C29—O7	0.3 (3)	C38—C31—N4—C37	163.3 (3)
C21—C28—C29—O7	−179.4 (3)	C38—C31—N4—C40	−30.4 (4)
C22—C28—C29—C24	178.9 (3)	C34—C40—N4—C37	−140.3 (2)
C21—C28—C29—C24	−0.8 (5)	C35—C40—N4—C37	−16.1 (3)
O9—C24—C29—C28	131.2 (3)	C34—C40—N4—C31	52.5 (4)
C30—C24—C29—C28	13.9 (4)	C35—C40—N4—C31	176.7 (3)
O9—C24—C29—O7	−50.3 (4)	C8—C9—O1—C3	−1.3 (3)
C30—C24—C29—O7	−167.6 (2)	C4—C9—O1—C3	179.2 (3)
O9—C24—C30—N3	−158.5 (2)	C2—C3—O1—C9	1.5 (4)
C29—C24—C30—N3	−36.1 (3)	C18—C19—O4—C13	−0.9 (3)
O9—C24—C30—C25	83.4 (3)	C14—C19—O4—C13	−179.0 (3)
C29—C24—C30—C25	−154.2 (2)	C12—C13—O4—C19	0.9 (4)
C26—C25—C30—N3	11.5 (3)	C28—C29—O7—C23	−0.2 (3)
C26—C25—C30—C24	134.6 (3)	C24—C29—O7—C23	−178.9 (3)
C38—C32—C33—O10	0.4 (4)	C22—C23—O7—C29	0.0 (3)
C40—C35—C36—C37	−16.3 (4)	C38—C39—O10—C33	−0.7 (3)
C35—C36—C37—O11	−174.2 (3)	C34—C39—O10—C33	−178.4 (3)
C35—C36—C37—N4	7.0 (4)	C32—C33—O10—C39	0.1 (4)

### Hydrogen-bond geometry (Å, º)

**Table:**

*D*—H···*A*	*D*—H	H···*A*	*D*···*A*	*D*—H···*A*
O3—H3*O*···O2^i^	0.84 (2)	1.90 (2)	2.681 (2)	154 (3)
O6—H6*O*···O8^ii^	0.86 (2)	1.93 (2)	2.766 (3)	166 (4)
O9—H9*O*···O5^ii^	0.85 (2)	1.92 (2)	2.737 (3)	161 (3)
O12—H12*O*···O11^iii^	0.83 (2)	1.98 (2)	2.797 (3)	167 (3)

Symmetry codes: (i) −*x*+1/2, −*y*, *z*−1/2; (ii) −*x*+3/2, −*y*+1, *z*−1/2; (iii) −*x*+3/2, −*y*+2, *z*+1/2.
